# The NFκB signaling system in the generation of B-cell subsets: from germinal center B cells to memory B cells and plasma cells

**DOI:** 10.3389/fimmu.2023.1185597

**Published:** 2023-12-11

**Authors:** Koushik Roy, Mainak Chakraborty, Ashok Kumar, Asit Kumar Manna, Neeladri Sekhar Roy

**Affiliations:** ^1^ Division of Microbiology and Immunology, Department of Pathology, School of Medicine, University of Utah, Salt Lake City, UT, United States; ^2^ Division of Immunology, Indian Council of Medical Research-National Institute of Cholera and Enteric Diseases, Kolkata, India; ^3^ Huntsman Cancer Institute, University of Utah School of Medicine, Salt Lake City, UT, United States; ^4^ Department of Biochemistry, School of Medicine, Emory University, Atlanta, GA, United States

**Keywords:** B cell, memory B cell, plasma cell, NFκB, and cell signaling

## Abstract

Memory B cells and antibody-secreting cells are the two prime effector B cell populations that drive infection- and vaccine-induced long-term antibody-mediated immunity. The antibody-mediated immunity mostly relies on the formation of specialized structures within secondary lymphoid organs, called germinal centers (GCs), that facilitate the interactions between B cells, T cells, and antigen-presenting cells. Antigen-activated B cells may proliferate and differentiate into GC-independent plasmablasts and memory B cells or differentiate into GC B cells. The GC B cells undergo proliferation coupled to somatic hypermutation of their immunoglobulin genes for antibody affinity maturation. Subsequently, affinity mature GC B cells differentiate into GC-dependent plasma cells and memory B cells. Here, we review how the NFκB signaling system controls B cell proliferation and the generation of GC B cells, plasmablasts/plasma cells, and memory B cells. We also identify and discuss some important unanswered questions in this connection.

## Introduction

Following an infection or vaccination, secondary lymphoid organs undergo profound structural changes to form extrafollicular foci and germinal centers (GCs) (1, 2). Antigen-activated B cells within extrafollicular foci proliferate and differentiate into GC-independent plasmablasts (PBs), which generate short-lived immunity and memory B cells (MBCs) (1, 2). Antigen-activated B cells that enter the GC proliferate and undergo somatic hypermutation (SHM) of the B cell receptor (BCR) with an average of 10^-3^ mutations per base pair in each proliferative cycle to enhance affinity for antigens (3–5). High-affinity B cells capture more antigen than low-affinity B cells, present the antigen to T cells, and subsequently receive strong T cell help. GC B cells that receive strong T cell help (mediated through the interaction of the CD40 receptor on GC B cell and the CD40 ligand on T cell) become affinity mature and differentiate into long-lived plasma cells (PCs). GC B cells that receive weak T cell help (weak activation through CD40) differentiate into MBCs, while others receiving a little/no T cell help undergo apoptosis (1, 2). However, this affinity-based selection model of PCs and MBCs generation has recently been challenged (1–5). Our single-cell lineage tracking study found that B cells show cell-to-cell variability in their proliferative capacity in response to BCR-independent stimulation, even when they express the same BCR (HEL transgenic BCR) (6). Both computational modeling and experimental results show that variable proliferative capacity is due to preexisting variation in the molecular networks, which is independent of BCR affinity. Hence, the selection of GC B cells may be a combinatorial effect of BCR affinity, preexisting variation in the molecular networks of the GC B cells during recruitment to the GC, and the complex environment of GC itself.

Mice deficient in T cells produce class-switched IgG antibodies upon viral infection and T cell-independent immunization (7, 8). A recent study has shown that “TLR-BCR linked co-engagement” with TLR-ligand and antigen generates T cell-independent class-switched and hypermutated high-affinity antibodies and GC-like structure (9). Another genetic fate mapping study shows that T cell-independent immunization develops transient GCs and generates GC-derived PCs and MBCs (10). Thus, both T cell-dependent and independent pathways generate GC and high-affinity antibodies, though the T cell-dependent pathway generates GC and high-affinity antibodies more efficiently.

GC B cells circulate between the two distinct anatomical zones of GC viz the light zone (LZ) and the dark zone (DZ). GC B cells undergo rapid proliferation (6-8 h) and SHM within the DZ to acquire affinity-improving mutations and return to the LZ, where they are tested for antigen affinity and the affinity-damaging mutation lead to apoptosis (2, 11, 12). Affinity-matured GC B cells differentiate into PCs within the LZ (2). Whether the generation of MBCs requires affinity maturation or not is controversial (1, 2, 5). The diversity and affinity of antibodies generated in response to an immune challenge are largely GC-dependent. The success of vaccination and protection from re-infection depends on the longevity of the generated antibodies and MBCs. As a result, GC B cells play a key role in generating long-lasting protective humoral immunity. However, chronic infection and other pathological conditions may disrupt GC B cell differentiation and contribute to lymphoid malignancy and autoimmunity (13). Therefore, precise regulation of GC B cell differentiation is needed to generate effective humoral immunity without generating B cell lymphoma/autoimmunity. The accurate regulation of GC B cell differentiation is controlled by the coordination of cell signaling pathways (such as NFκB, PI3K/AKT, MAPK, and STAT) and transcription factors (such as NFκB, IRF, Myc, Bcl-6, OCA-B, Bach2, etc.) (1, 14). The transcription factor NFκB is a direct stimulus-responsive transcription factor. Stimulation leads to nuclear translocation of NFκB within a few minutes to an hour and activates the transcription of many key regulators essential for GC B cells, PCs, and MBCs (1, 14–21) (discussed below). It has been shown that NFκB and its upstream signaling (defined here as the NFκB signaling system) are dysregulated in many B cell lymphomas and immune disorders (13, 22–25).

## Overview of the NFκB signaling system

In mammals, the transcription factor NFκB family comprises homo- and heterodimers formed combinatorially by five Rel family proteins RelA/p65, cRel, RelB, p50 (NFκB1), and p52 (NFκB2) (26–28). The five NFκB monomers can theoretically generate 15 possible dimers (29, 30). The three Rel family members, RelA, cRel, and RelB, have a DNA binding domain and function as transcriptional activators (30–33). The other two Rel family members, NFκB1 and NFκB2 have a DNA binding and an ankyrin repeat domain (ARD) (30, 33, 34). The ARD of NFκB1 and NFκB2 inhibits the activation of NFκB. Constitutive or stimulus-responsive proteolytic cleavage of the ARD generates p50 and p52 from NFκB1 and NFκB2, respectively. p50 and p52 contain a DNA binding domain but not a transcription activation domain and may inhibit transcription as homodimers (p50:p50 and p52:p52) (26, 29, 35, 36). However, p50:p50 and p52:p52 dimers may form a complex with co-activators (e.g., Bcl3 and IκBζ) to activate transcription (37, 38). The detail of NFκB signaling has been extensively studied and summarized in several excellent reviews (29, 39, 40). Here, we have briefly described the NFκB signaling system, primarily in the context of B-cells.

**Figure 1:**
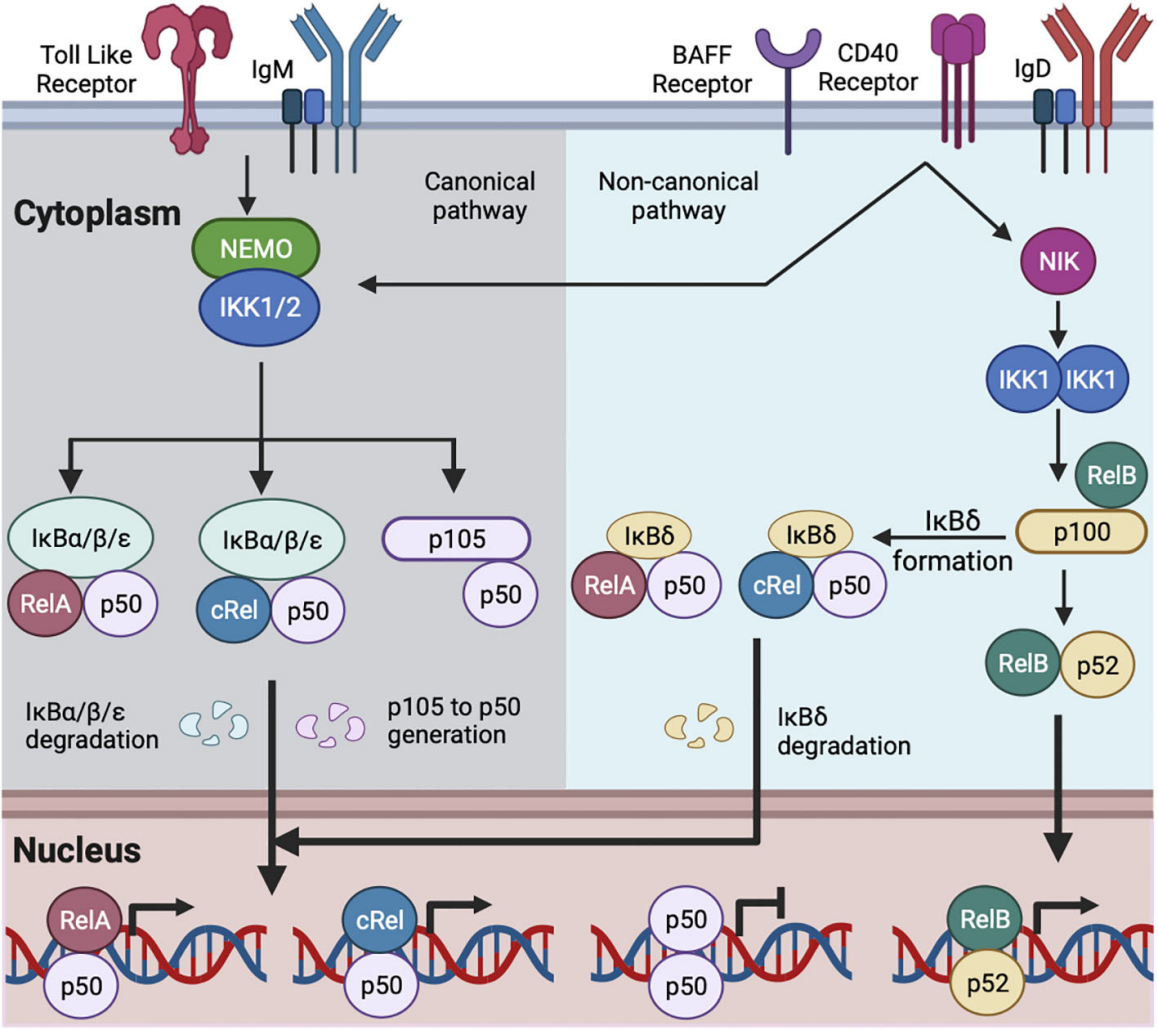
Schematic of canonical and non-canonical NFκB activation in B cell. TLR and IgM-mediated BCR signaling activate the canonical NFκB pathway (15, 35). IgD-mediated BCR signaling may activate both the canonical and non-canonical NFκB pathway (41, 42). CD40 and BAFF activate the canonical and non-canonical NFκB pathways (15). The canonical signaling activates NEMO and IKK1/2 containing complex. The activated IKK1/2 phosphorylates members of the IκBs (IκBα, IκBβ, and IκBε here referred as IκBα/β/ε) bound with NFκB, leading to the degradation of IκBα/β/ε. The degradation of IκBα/β/ε releases IκB-bound NFκB, which translocates to the nucleus. The activated IKK1/2 phosphorylates IκB-like molecule p105, and ubiquitin-mediated degradation of p105 generates p50 with the formation of RelA:p50, cRel:p50 and p50:p50 (15, 22, 35). RelA:p50 and cRel:p50 dimers are transcriptional activators. p50:p50 dimer may function as a transcriptional inhibitor and are present in naïve mature B cell (35). B cell activation by canonical pathway replaces p50:p50 with RelA:p50 and cRel:p50 (15, 35). The non-canonical signaling stabilizes NIK and subsequent activation of IKK1. The activated IKK1 phosphorylates IκB-like molecule p100 and generates p52. The degradation of RelB-bound p100 generates RelB:p52 dimer, and its nuclear translocation (15). The multimeric association of p100 generates IκBδ. IκBδ remains predominantly bound with cRel:p50 and RelA:p50 dimers. The activated IKK1 degrades IκBδ and releases cRel:p50 and RelA:p50 dimers to the nucleus (16, 43).

In the absence of extra-cellular stimuli, NFκB is associated with inhibitors of NFκB (IκBα, IκBβ, IκBε, and IκBsome) in the cytosol. The activation of B cells by exogenous (foreign materials: e.g., protein/peptide antigen, LPS, etc.) and endogenous (host-derived materials: e.g., CD40-ligand, BAFF, etc.) stimuli causes degradation of IκBs by proteolysis and releases IκB-bound NFκB dimers for their translocation to the nucleus where they activate transcription (15) (**Figure 1**) (details below). Naïve B cells are enriched for nuclear p50:p50 homodimer, which may function as a transcriptional inhibitor; B cell activation replaces inhibitor NFκB (p50:p50) with activator NFκB (cRel:p50/RelA:p50) (35, 44). IgM-mediated BCR signaling and TLR signaling in B cells predominantly activate RelA:p50, cRel:p50, and p50:p50 through the canonical pathway (15–18, 45–47). IgD-mediated BCR signaling induces expression of NFκB2 and generates p52, suggesting activation of the non-canonical NFκB pathway (41). CD40 signaling in B cells activates both canonical and non-canonical pathways, leading to nuclear translocation of RelA:p50, cRel:p50, and RelB:p52 (15, 48, 49). BAFF signaling alone predominantly activates the non-canonical pathway more than the canonical one (15, 50, 51) (**Figure 1**).

Canonical NFκB signaling is transduced by a NEMO-dependent kinase (IKK) complex composed of IKK1, IKK2, and NFκB essential modulator (NEMO). The activation of this IKK complex is NEMO-dependent and mediated by phosphorylation-dependent activation of IKK2 (29, 52, 53). The activated IKK2 phosphorylates IκBα, IκBβ, and IκBε, leading to their degradation and freeing NFκB dimers for nuclear translocation (26–28). The canonical NFκB signaling pathway in B cells predominantly activates RelA:p50, cRel:p50, and p50:p50 dimers (15–17, 35). Non-canonical NFκB signals are transduced in a NEMO-independent but NIK (NFκB inducing kinase) and IKK1-dependent manner (29, 52). The non-canonical pathway has dual functions. The first function is to process the p100 monomer to p52, leading to the formation of RelB:p52 dimer (43, 54). Unprocessed p100 oligomerizes and forms the IκBsome inhibitory complex (55). The second function is to degrade the IκBsome and release IκBsome bound NFκB, including RelA:p50 and cRel:p50 (16, 43). The non-canonical NFκB in B cells predominantly activates RelB:p52; however, in a context-dependent (e.g., anti-IgM and BAFF co-stimulation, discussed below) and cell type-specific manner, the non-canonical pathway also activates RelA:p50, and cRel:p50 dimers (15, 16, 56).

## The NFκB signaling system in B cell proliferation and survival

**Figure 2:**
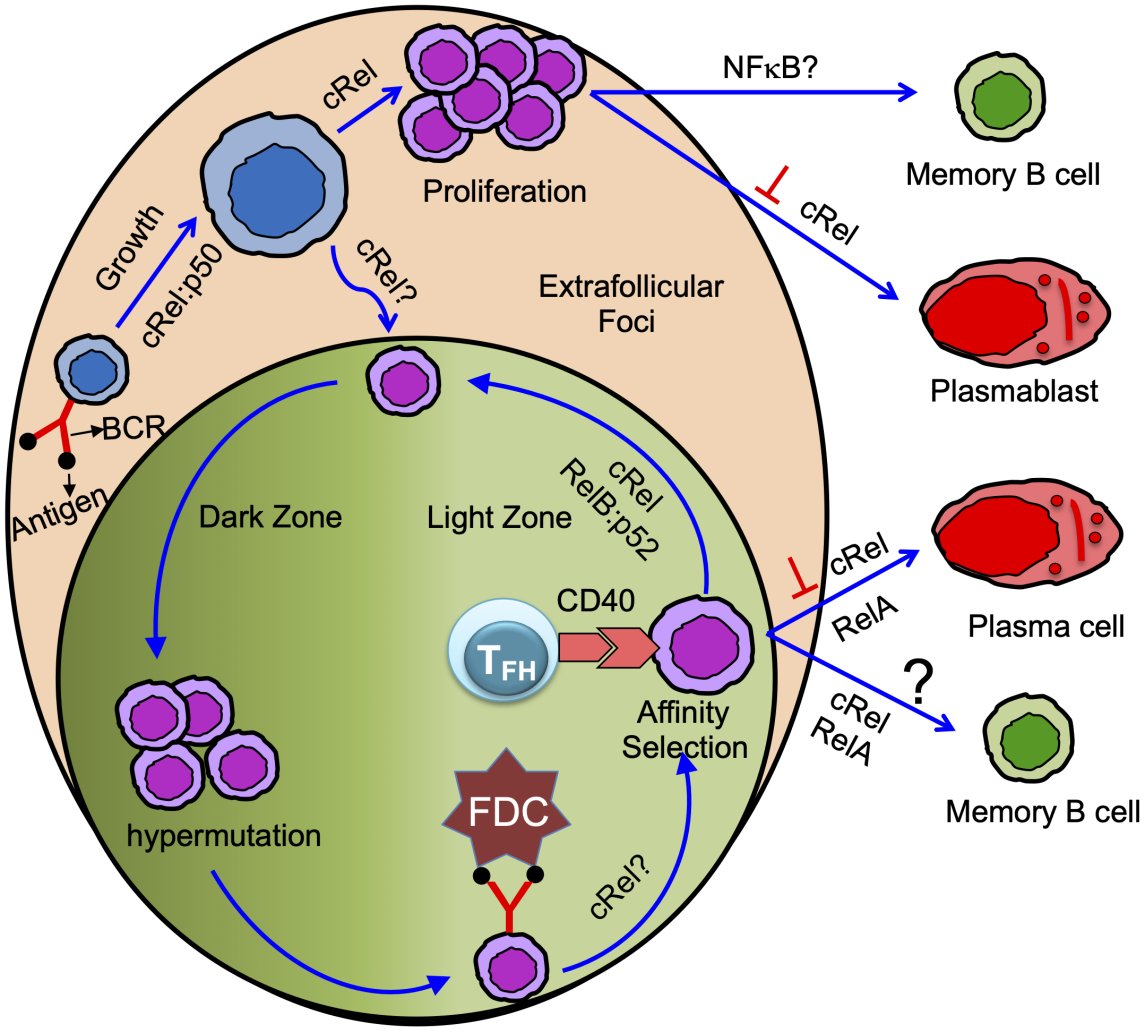
Antigen-specific naïve B cells following antigen binding activate and grow in size. cRel:p50 dimer control activation/B cell growth (58, 59). Activated B cells proliferate in the extrafollicular foci or differentiate in GC B cells. cRel is required for B cell proliferation (15, 58, 60, 61). Whether cRel is required in B cells for GC initiation or formation is not yet clear (62). Proliferating B cells in the extrafollicular foci differentiated into memory B cells and plasmablast. cRel inhibits plasmablast differentiation (21), and BAFF signaling needed for GC-independent memory B cell generation suggests NFκB could control GC-independent memory B cell generation (63). GC has anatomically two distinct zones: dark and light zone. Activated B cells that enter GC and differentiate into GC B cells undergo proliferation coupled with somatic hypermutation in the dark zone. GC B cells in the dark zone rapidly proliferate and undergo somatic hypermutation of B cell receptors. GC B cells in the light zone acquire antigen from follicular dendritic cell (FDC). Light zone B cells present the antigen to the T follicular helper (T_FH_) cell and receive T cell help mediated by CD40 signaling (Note: T_FH_ cell also provides other modes of help such as IL21, IL4, etc. Here, we emphasize only the CD40 signal). Long-lived plasma cells are generated from affinity-selected GC B cells. It is controversial whether affinity-based selection is required for the generation of GC-derived memory B cells. cRel is required for GC maintenance and likely control the light to dark zone transition (20). cRel may control BCR-mediated GC survival as BCR-activated light zone B cells show NFκB target gene expression (64). RelA is required, but cRel inhibits, for GC-derived plasma cell generation (20). RelB:p52 is required for cell cycle entry of GC B cells and likely control the interaction of GC B cells with T_FH_ cells (19). Thus, RelB:p52 control re-entry of GC B cells from light to dark zone. Both cRel and RelA control GC-derived memory B cell generation (65), and the conclusion is based on an induced GC-B cells culture system.

Naïve B cells are activated by antigen/ligand binding to cell-surface receptors, e.g., BCR signaling is activated by an antigen, TLR signaling is activated by TLR-ligand (e.g., LPS, CpG, etc.), CD40 receptor signaling is activated by CD40-ligand, and BAFF receptor signaling is activated by BAFF (**Figure 1**). BCR, CD40, and TLR signaling- all result in B cell activation, proliferation, and survival, while BAFF signaling, without co-stimulation, results in B cell survival (15, 22, 57). These signals activate NFκB, as discussed above (**Figure 1**). Activated B cells enter the growth phase and increase in cell size, and during the growth phase, they are protected from cell death (**Figure 2**) (60, 66). It has been shown that mature B cells stimulated for 24 hour activate the proliferative program, and these activated B cells are programmed to divide multiple times without further stimulation, suggesting that induction of key regulators within the first few hours may control division number (67). In line with this, Heinzel et al. showed that Myc expression before the 1^st^ division determines the maximum division number (68). Myc is a cRel target gene (58). Therefore, it is possible, but as yet unproven, that the extent of NFκB activation before 1^st^ division can control the maximum division number.

IKK2 deficiency leads to diminished NFκB activation. IKK2-deficient B cells show reduced mature B cell numbers and impaired B cell activation/proliferation upon mitogenic stimulation with LPS, anti-IgM, or anti-CD40 (69). NEMO-deficient B cells also show reduced NFκB activation and reduced generation of mature B cells, and the phenotype is similar to IKK2 deficiency (69). Constitutive activation of the canonical NFκB pathway, using constitutively active IKK2 (IKK2ca mutant), enhances B cell survival, leading to B cell hyperplasia (70). NIK-inactivating mutations impair the non-canonical NFκB pathway, leading to reduced p100 degradation and reduced generation of p52 (71, 72). It has been shown that NIK-inactivating mutant mice (aly/aly mice) have reduced B cell proliferation following LPS and anti-CD40 stimulation (71, 72), suggesting NIK activity is required for B cell proliferation. The activation of canonical NFκB results in the degradation of IκBα and IκBε, and the activation of non-canonical NFκB results in the degradation of IκBsome (IκBδ, p100 oligomer) (**Figure 1**). It has been shown that individual knockout of IκBα and IκBε enhances B cells proliferation and survival upon LPS and anti-IgM stimulation (17, 73, 74). Similarly, IκBsome reduction, caused by NFκB2 heterozygosity, enhances B cell proliferation and survival upon anti-IgM stimulation (16). Thus, hyper NFκB activation enhances B cell proliferation and survival. Both the canonical and non-canonical NFκB pathways are required for complete mitogen-induced B cell proliferation and survival.

All mitogens that activate the canonical NFκB pathway in B cells need cRel for proliferation, although the extent of cRel dependency varies, IgM-mediated signaling more dependent on cRel than LPS (**Figure 2**) (46). IgD-mediated signaling generates p52, although it fails to activate p65 (41). B cells deficient in NFκB2 show moderate defects in B cell proliferation in response to IgD signaling (75). B cells double deficient in p65 and p50 (p65-/-p50-/-) show impaired proliferation in response to IgD signaling, although B cells deficient in either p65 or p50 have a little/no proliferative defects (42). Tonic BCR signaling mediated canonical pathway (cRel/RelA) activation is required to induce NFκB2 (16, 76). Thus, IgD signaling may activate both canonical and non-canonical NFκB (41, 42). NFκB1 is crucial for TLR4-dependent B cell proliferation (46, 77), presumably by enabling Tpl2-MAPK signaling (35). cRel deficient B cells show reduced expression of transcription factor IRF4, which is required for B cell proliferation (20, 78, 79). Although cRel deficient B cells still grow (enter the G1 phase), their transition from the G1 to S phase of the cell cycle is impaired (58, 60, 61). The failure to transit from the G1 to S phase has been attributed to impaired induction of transcription factor E2F3, which is required for G1 to S phase transition (80). Further, cRel-deficient B cells fail to upregulate the standard metabolic program associated with cell growth (20). The transcription factor Myc is required for B cell growth (81). cRel and NFκB1 double deficient B cells failed to grow in size (**Figure 2**) and have reduced Myc expression, suggesting both cRel and NFκB1 are required for G0 to G1 transition (58, 59). Transgenic Myc expression rescues B cell growth defects in cRel and NFκB1 double deficient B cells, though restoring Myc activity failed to drive proliferation upon stimulation (58). Therefore, NFκB plays multiple roles in the different phases of the cell cycle, and each NFκB subunit has distinct functions. cRel deficient B cells, upon BCR stimulation, failed to upregulate pro-survival regulators BclA1 and Bcl-xL, and Bcl2 transgenic expression inhibits BCR-induced cell death (80). Both cRel and NFκB1 are required to protect TLR4-stimulated B cells from apoptosis by blocking proapoptotic protein Bim (35, 82).

## The NFκB signaling system in isotype switching

Immunoglobulin is also known as BCR when present on the cell surface. Class switching shifts immunoglobulin class, for example, from the isotype IgM to IgG. Naïve B cells express IgM and IgD (41). Class switching produces multiple isotypes of antibodies with the same variable domains but differing in the constant domains of heavy chains (83). LPS activates NFκB and promotes class switching to IgG3, while CD40L+IL4 promotes class switching to IgG1 and IgE, suggesting both canonical and non-canonical NFκB pathways could control class switching (84, 85). The deletion of NFκB1 in B cells, or the transactivation domain of cRel in B cells, leads to defects in the transcription of heavy chain constant region (86, 87). Class switch recombination (CSR) occurs within the region of the repeat sequence of the constant region, and mitogen-activated NFκB promotes transcription of the repeat sequence, thereby promoting isotype switching (88–90). It has also been shown that isotype switching depends on NFκB binding to the 3’ IgH enhancer region (91, 92). CSR strictly depends on activation-induced cytidine deaminase (AID) (93–95). AID is also required for SHM (details below). AID expression must be tightly controlled as AID-mediated off-target activity poses a serious risk to the genome integrity and translocations, mutations, and oncogenesis (96). NFκB signaling is a key inducer of AID, mediated by the co-activation of TLR and BCR and by the interaction of CD40 receptor and ligand (41, 97). At least during CSR, p52 and RelA are recruited to the promoter and upstream enhancer regions of the *AICDA* gene, respectively, which encodes AID (98). Co-factors are also involved in NFκB-mediated AID activation, including HoxC4, SP1, and SP3 (93, 99). Xu et al. have shown that radiation-sensitive 52 (Rad52) is required to mediate IgD class switching through the downregulation of ZFP318, and Rad52 phosphorylation is strongly linked with high levels of IgD autoantibodies in mice models of lupus as well as SLE patients (100).

Cytokines released by T cells, such as IL-4 and TGF-β, act as secondary inducing stimuli directing isotype switching (101). Cytokines are crucial for class switching; for instance, IL-4 causes IgG1 and IgE synthesis (102, 103), while TGF-β causes IgA class switching (104, 105). The induction of the T cell-dependent IgA class switch requires TFG-β and CD40 ligand (106–108), while T cell-independent IgA class switch requires LPS along with TFG-β or BAFF and APRIL produced by DCs (108–110). CD40, LPS, and BAFF activate NFκB, suggesting NFκB could be essential for the IgA class switch. Mice lacking NIK produce less homeostatic IgA and exhibit defective SHM (111–113) and reduced synthesis of antigen-specific antibodies (72, 111, 114). Patients with B cell lymphopenia, who experience lower frequencies of class-switched MBCs and hypogammaglobulinemia, are frequently shown to carry a biallelic mutation of NIK (115). BAFF and APRIL promote the binding of MyD88 to TACI, which is necessary to activate NFκB and induce AID to promote CSR (116). Therefore, the picture emerges that NFκB is essential for CSR by directly controlling the transcription of immunoglobulin and then in an indirect way by controlling the transcription of AID.

## The NFκB signaling system in germinal center B cells and somatic hypermutation

BCR functions as both a signaling molecule and an endocytic receptor to capture antigens for T cell help. BCR signaling in GC B cells is short-lived and attenuated by high phosphatase activity (117, 118). A recent study showed that IgA BCR transduces stronger BCR signaling than IgM BCR in intestine-generated GC B cells, and IgA BCR signaling is required for GC B cell survival (119). In line with this, BCR signaling in GC B cells has been shown to prolong survival and thus primes for selection (120). IgM BCR signaling fails to induce nuclear translocation of NFκB in GC B cells, although it induces nuclear translocation of NFκB in mature B cells (121, 122). CD40 signaling induces nuclear translocation of NFκB in both GC B cells and mature B cells (15, 121). However, a recent study revealed that both BCR and CD40 signaling induce the expression of NFκB target genes (such as *nfkbia* and *nfkbie*) in human tonsillar GC B cells, though the amplitude of NFκB target gene expression is much higher with CD40 signaling than BCR signaling (64).

A constitutively active IKK2 (IKK2ca mutant) leads to elevated constitutive NFκB activity and shows enhanced B cell survival. However, immunization of the IKK2ca mouse results in reduced GC B cells, although PC numbers and antibody production remain unaltered (123). Deletion of Blimp1 (a master regulator of PC differentiation) in IKK2ca mice enhances the generation of GC B cells upon immunization but reduces the generation of PCs. Interestingly, IKK2ca mice develop PC hyperplasia at an older age, and deletion of Blimp1 in IKK2ca mutant mice leads to the development of activated B cell-like diffuse large B cell lymphoma (123). Similarly, adoptive transfer of IκBα knockout fetal liver cells (which have elevated constitutive NFκB activity) and subsequent immunization of the recipient mice results in impaired GC formation (73). Conversely, IκBε knockout enhances the generation of GC B cells (124). IκBα and IκBε inhibit cRel and RelA differentially, suggesting cRel and RelA could have distinct roles in controlling GC B cell formation (17, 125). Further, a recent study identified cRel- and RelA-specific target genes in BCR-stimulated B cells and found new cRel-specific target genes (Hhex/Bcl6b) that are known to play a critical role in GC B cells (126).

It is well established that cRel and RelA are critical for physiological B cell responses, and their misregulation leads to B cell-mediated diseases such as immune deficiencies, B cell lymphoma, and autoimmune disorders (13, 127). Mice with conditional deletion of cRel in B cells (CD19-Cre), upon TD immunization, fail to generate GC B cells 5 days after TD immunization (62), suggesting cRel is required for GC formation/initiation (**Figure 2**). Mice with conditional deletion of cRel in GC B cell (Cγ1-Cre), upon TD immunization, develop GCs (day 7, when the GC consists of predominantly DZ cells), which then start to involute and collapse (at 14 days). This study suggests that cRel in GC B cells is not required for the DZ establishment but is required for GC maintenance either by facilitating the recirculation of LZ to DZ or by priming LZ B cells through BCR signaling (**Figure 2**) (20). cRel-deficient B cells are known to have defective survival, and Bcl2 transgenic expression blocks BCR-induced cell death in cRel-deficient B cells (80). Therefore, it was anticipated that cRel-deficient GC B cells failed to maintain the GC due to impaired survival of GC B cells. However, cRel-deficient GC B cells do not show impaired expression of survival regulators (Bcl2, Bcl2L1, and Mcl1), and importantly, cRel-deficient GC B cells expressing the Bcl2-transgene fail to rescue GC collapse, suggesting GC collapse is not due to impaired survival in cRel deficiency. Interestingly, cRel-deficient GC B cells fail to upregulate the metabolic programming required for B cell growth, suggesting that cRel-dependent B cell growth could cause GC collapse (20). Myc is required for B cell growth (58). cRel-deficient B cells reduce Myc target gene expression signature (58), and cRel overexpression upregulates it (24), suggesting that Myc induction is cRel-dependent. It would be interesting to test whether transgenic expression of Myc in cRel-deficient GC B cells could rescue GC collapse. These effects are predominantly cRel-specific as RelA deficiency is associated with unaltered GC formation (20).

NFκB1 p105 has two functions. The first function is that the N-terminal domain of p105 generates p50, which forms a dimer with other NFκB family monomers, and the second function is that the C-terminal domain of p105 functions as an IκB and inhibits activation of NFκB (128) and Tpl2-MAPK signaling (35). Canonical pathway activation leads to proteolysis of the C-terminal domain of p105 and the formation of p50 hetero- or homodimers (129). To investigate the effect of p105 proteolysis on the GC and TD-dependent antibody production, Jacque et al. studied a signal-induced proteolysis-resistant mutant of p105 (*NFkB1* ^SSAA^, mutation of NFκB1 in the IKK2-target serine to alanine) which shows a block in p50 formation but retain a dominant IκB function (130). *NFkB1* ^SSAA^ B cells show reduced nuclear p50, RelA, and cRel, whereas an unaltered level of RelB and p52 upon CD40 stimulation, suggesting *NFkB1* ^SSAA^ is deficient in canonical NFκB activation but likely not in non-canonical NFκB activation. *NFkB1* ^SSAA^ mice have a normal number of follicular B cells, although the number of marginal zone B cells is reduced. *NFkB1* ^SSAA^ follicular B cells show impaired survival and proliferation upon IgM and CD40 stimulation. The TD immunization of *NFkB1* ^SSAA^ mice shows reduced antigen-specific GC B cell formation and antibody production. Interestingly, increasing p50 levels in *NFkB1* ^SSAA^ mice restores antigen-specific GC B cell and antibody generation upon TD immunization (130). The increased survival of *NFkB1* ^SSAA^ B cells by Bcl-XL overexpression was unable to rescue TD antibody production. Therefore, the above study suggests that p50 (created by the proteolysis of p105) has multilayer functions in generating GC B cells and antibody production, beyond the role of p50-containing dimers in increasing B cell survival and proliferation.

NFκB2 (p100), similar to NFκB1, has two functions. The N-terminal domain of p100 generates p52, which predominantly forms a dimer with RelB (RelB:p52), and the C-terminal domain of p100 functions as an IκB (known as IκBδ) within the IκBsome and inhibits activation of NFκB (16, 43). Almaden et al. have shown that anti-IgM and BAFF co-stimulation leads to the degradation of IκBδ and enhances cRel activity with the subsequent enhancement of B cell proliferation (16). The authors have reduced the expression of IκBδ using NFκB2 heterozygosity, and the NFκB2 heterozygous B cells prolong stimulus-induced cRel activation and enhance B cell proliferation and antibody production upon TD immunization. The increased antibody production in NFκB2 heterozygosity could be due to increased GC formation. De-Silva et al. generated GC B cell-specific knockout of NFκB2 and RelB:p52 dimer to test the function of NFκB2 and RelB in GC B cell formation (19). NFκB2-deficient GC B cells show a partial defect in GC formation, though NFκB2 heterozygosity has no effect (19). Interestingly, the combined deficiency of NFκB2 and RelB in GC B cells led to the collapse of established GCs, whereas RelB deficiency alone shows no defect. However, precursor GC B cells in the peri-follicular region show higher RelB expression and nuclear translocation (131). The combined deficiency of NFκB2 and RelB in GC B cells results in reduced cell cycle entry and expression of Inducible T Cell Costimulator Ligand (ICOSL), which is required for the optimal interactions between B cells and T cells in the GC (**Figure 2**) (19). The increased antibody production in NFκB2 heterozygous mice could be due to the increased generation of antibody-producing cells from the GC-independent pathway. The above studies indicate that NFκB2 inhibits sustained cRel activation by forming IκBδ, thereby reducing B cell proliferation and antibody production, while NFκB2-derived p52 generates RelB:p52 dimer, promoting GC maintenance. Thus, NFκB2 seems to have two opposite functions in humoral immunity. It is possible that the inhibitory function of NFκB2 (mediated by IκBδ) controls the GC-independent response, while transcription factor NFκB2 (mediated by RelB:p52) controls the GC-dependent response.

GC B cells undergo SHM to improve the affinity of the antibody to the cognate antigen and become affinity mature. SHM involves programmed mutations in variable regions, while CSR modifies the constant region of immunoglobulin genes (132). SHM occurs in DZ of GC, and nuclear translocation of NFκB has only been observed in LZ but not in DZ GC B cells (19, 133). However, both SHM and CSR are controlled by AID, which itself is controlled by NFκB (discussed above). cRel-deficient GC B cells show reduced affinity maturation and SHM of GC B cells (20). However, transgenic cRel expression in GC B cells does not significantly affect affinity maturation and SHM (24). The reduced SHM in cRel-deficient GC B cells could be either due to impaired AID expression or GC collapse. RelA and p52 contribute to AID expression in mature B cells (98). RelA-deleted GC B cells undergo normal affinity maturation, suggesting that RelA is not required for affinity maturation (20). It is possible that RelA controls AID expression in mature B cells, which is critical for CSR but not in GC B cells. Alternatively, it is possible that cRel compensates for RelA in RelA deficient GC B cells and facilitates SHM. Further investigations are needed to determine the role of NFκB systems in SHM and affinity maturation.

## The NFκB signaling system in plasmablast/plasma cell development and survival

When stimulated by an antigen, activated B cells proliferate and differentiate into more specialized antibody-secreting cells. Antibody-secreting cells are generated by T cell-dependent and independent immunization and are heterogeneous in terms of their origin, secretory function, and lifespan (134). Antibody-secreting cells are broadly characterized in two types: PBs and PCs. PBs are cycling and short-lived antibody-producing cells, whereas PCs are terminally differentiated antibody-producing cells with life spans that can be short, long, or very long (135, 136). The gene regulatory network of short-lived PCs gradually changes to long-lived PCs over time (136–138). PCs reside in secondary lymphoid organs for a shorter duration and in the bone marrow for decades (139). A recent study showed that short-lived PCs were progressively differentiated into long-lived ones after arriving in bone marrow (140).

The expression of Blimp1, a master regulator for antibody-secreting cell generation, can distinguish cycling PBs and quiescent PCs. PBs express a low level of Blimp1, whereas PCs express a high level of Blimp1 in both mice and human (137, 141, 142). IRF4 is a key transcription factor for PC generation and enhances Blimp1 expression by creating a positive feedback loop with Blimp1 (143, 144). Both cRel and RelA induce IRF4 expression (21). RelA is required for Blimp1 expression and PC generation (**Figure 2**) (20, 145). RelA and IRF4 are induced during the early phase of B cell activation. However, activated B cells do not differentiate during early B cell activation, suggesting Blimp1 expression is inhibited during the early phase of B cell activation. It was not clear how Blimp1 expression was inhibited during B cell activation until Roy et al. discovered that cRel inhibits Blimp1 expression by Bach2 (21). It is well established that cRel promotes cell cycle progression, whereas Blimp1 inhibits cell cycle progression (59, 80, 146). Based on these observations and computational modeling of the molecular gene regulatory network, Roy et al. hypothesized that cRel inhibits Blimp1 expression. Indeed, cRel was found to be gradually downregulated from GC B cell> PB>PC, and the level of cRel expression was correlated with active cell cycle states (21). The expression of Blimp1 and cRel are inversely correlated, suggesting cRel downregulation may be a requirement for Blimp1 expression and PC generation. To determine whether cRel downregulation is a requirement to become PCs, cRel was overexpressed, and it was observed that cRel overexpression inhibits the generation of PCs by inhibiting Blimp1 expression, and cRel knockout enhances the generation of PCs and Blimp1 expression (**Figure 2**) (21). Further, Roy et al. investigated the mechanism of cRel downregulation in PCs and found that when Blimp1 was deleted, activated B cells failed to downregulate cRel. Mutation of Blimp1 binding site in cRel promoter impaired cRel downregulation, indicating that Blimp1 represses cRel by directly binding to cRel promoter (21). Our study showed that cRel inhibits PCs generation by repressing Blimp1, a RelA target gene, suggesting cRel and RelA antagonize B cell differentiation to PCs. A recent study also showed that functional antagonism of cRel and RelA in BCR stimulated B cells (126).

Studies have also revealed that human tonsillar PCs and precursor PCs in the GC express high levels of NFκB2 compared to other tonsillar lymphocyte populations (19). The deletion of NFκB2 leads to reduced antigen-specific antibody production in a mouse model (19, 147). NFκB2-deficient mice show IgA downregulation and significantly elevated IgM in the small intestine mucosa. The lamina propria of the small intestine of NFκB2 deficient mice had fewer CD138^+^ PCs that produced IgA (148). Almaden et al. showed that germline NFκB2 heterozygosity enhanced antibody production and proposed that NFκB2 heterozygosity leads to disruption of IκBδ and sustains cRel activity leading to enhance B cell proliferation and subsequent antibody production (16). Overall, the above studies suggest NFκB2 deficiency reduces antibody production, whereas NFκB2 heterozygosity enhances antibody production. The role of NFκB2 in these under-expression systems is likely a complex combination of the effects of p100 and p52. It is possible that the inhibitory function of NFκB2 (mediated by IκBδ) controls extrafollicular antibody production, whereas the transcription factor NFκB2 (mediated by RelB:p52) controls GC-dependent antibody production. The role of RelB:p52 in GC response is discussed above in detail.

PCs may not be naturally long-lived; their ability to access and interact with particular niches is essential to their survival. Specialized bone marrow niches support the survival of PCs by producing APRIL, BAFF, IL-6, CD44, and CXCL12 (149). PCs upregulate the expression of cell surface receptor BCMA, which provides survival signals upon binding with APRIL and BAFF (150). Both BAFF and APRIL activate NFκB signaling (151). T cell costimulatory receptor type CD28 is also essential for PC survival (152). The authors showed that CD28 selectively transmits pro-survival signaling to PCs. Reactive oxygen species (ROS) generation, mitochondrial mass/respiration, and glucose absorption were all elevated by CD28 signaling in PCs. In PCs, CD28 activation elevates the NFκB target gene IRF4, and IRF4 levels are associated with glucose absorption, mitochondrial mass, ROS, and CD28-mediated survival. Multiple myeloma, a plasma cell cancer, shows constitutive activation of both canonical and non-canonical NFκB pathways. The growth and survival of a subset of multiple myeloma depends on RelA alone, suggesting a RelA-mediated gene expression program could be critical for PC survival (13). Another study identified that tumor-promoting cytokines, such as tumor necrosis factor, activates RelB:p50 in multiple myeloma cell line. RelB:p50 is necessary and sufficient to provide pro-survival and anti-apoptotic signals in multiple myeloma (153). Inhibition of NIK results in apoptosis in multiple myeloma cells through reduced expression of anti-apoptotic proteins Bcl2L1, Bcl2A1, and Mcl1 (154). Overall, the NFκB pathway seems to play an important part in creating favorable conditions for PC survival, and the requirement of RelA/RelB in PC survival seems context-dependent.

## The NFκB signaling system in memory B cells

MBCs develop both GC-dependent and -independent pathways. They constitute an essential part of the adaptive immune system as they circulate in the bloodstream for an extended time (155). MBCs remember the antigen and unleash a stronger secondary immune response upon exposure to the same antigen later in life (156). MBCs could mutate their immunoglobulin gene, differentiate into antibody-secreting cells, and produce an antibody with altered antigen specificity and affinity. Therefore, MBCs could protect against the same pathogen as well as antigen-drifted pathogens such as COVID-19 and influenza (157, 158). Despite the outstanding success of some vaccines, not all generate long-lasting humoral immunity; for example, influenza and COVID-19 vaccines require periodic administration (159). The vaccine goal for a highly mutating pathogen (e.g., influenza, SARS-Cov-2) is to generate higher numbers of MBCs. Influenza vaccine effectiveness drops even within a season due to both short-lived antibody production and higher antigen drift of the influenza virus (159, 160). Influenza vaccine development aims to produce more MBCs (159).

MBCs are comprised of phenotypically distinct MBC subsets with specialized functions. MBCs are present in the blood, lymphoid organs (e.g., tonsils), and barrier tissues, including the gut, lungs, and skin, in both human and mice (161, 162). MBC subsets can be characterized based on the expression of BCR isotypes, unswitched IgM/IgD MBCs, and switched IgG, IgE, and IgA MBCs. IgG transduces stronger BCR signaling than IgM in MBCs; thus, IgG lowers the activation threshold of MBC and enhances the propensity of PC generation than IgM (163). Antibody isotype-independent MBC subsets are characterized by differential expression levels of PDL2 and CD80 in mice. PDL2+CD80+ MBCs preferentially differentiate into PCs upon rechallenge, and PDL2-CD80- MBCs preferentially seed in the GC (156, 164). Therefore, MBCs are reactivated by both BCR intrinsic and extrinsic pathways. Human MBCs can be identified based on the expression of CD27, a marker of antigen-experienced B cells (156, 165, 166). Interestingly, the number of human MBCs (CD27+ B cells) is higher than naïve B cells in the peripheral blood of aged individuals (167). Moroney et al. have identified the proportion of different human MBC subsets; IgD+CD27+ MBCs are about 10%, IgG+CD27+ MBCs are about 6.5%, and IgA+CD27+ MBCs are about 5% of total CD19+ B cells present in the peripheral blood of healthy human subject. The transcriptional signature of IgG+CD27+ and IgA+CD27+ MBCs are distinct from naïve B cells (165).

Lau et al. showed that B cell-intrinsic BAFF/BAFFR signaling is required for the GC-independent MBC generation, though BAFF/BAFFR signaling is not required for the GC-dependent MBC generation (63). BAFF is required for the survival of naïve mature B cells. The role of BAFF in MBC survival was unclear until Muller-Winkler et al. used a genetic knockout BAFF/BAFFR mouse model to study the function of BAFF/BAFFR signaling on the survival of MBCs (168). The authors found that knockout of BAFF/BAFFR leads to the loss of MBCs, and BAFF depletion by anti-BAFF monoclonal antibody treatment reduces lung-resident influenza-specific MBCs. BAFF predominantly activates the non-canonical (IKK1) NFκB pathway, though, under certain circumstances, it activates the canonical (IKK2) pathway. The author shows that IKK1 is partially required for IgM+ MBCs survival, and IKK2 is required for the survival of both IgM+ and IgG1+ MBCs. The combined BAFF and BCR signaling activates cRel in mature B cells (16). Studies have shown that RelB deficiency in humans results in impairment of B cell development, with an absence of CD27+ MBCs leading to severe B cell immunodeficiency and shortage in the secretion of antibodies (169). Overall, BAFF is required for GC-independent MBC generation, and MBC survival depends on the synergy of BCR- and BAFF-mediated activation of the NFκB pathway (170).

A recent study shows that CD40 signaling controls the generation of phenotypically defined MBCs, namely CD80^hi^ and CD80^lo^ MBC (65). A low CD40 signal leads to the generation of CD80^lo^ MBCs, and a relatively high CD40 signal leads to the generation of CD80^hi^ MBCs. CD40 signaling in GC B cells leads to the activation of cRel and RelA (121). Knockdown of cRel or RelA in “induced GC B cells” reduces the generation of CD80^hi^ MBC (**Figure 2**). NFκB activation may depend on the dose of CD40. A high CD40 signal activates NFκB and promotes the generation of CD80^hi^ MBCs, whereas a low CD40 signal fails to activate NFκB and promotes the generation of CD80^lo^ MBCs. CD40 activates both canonical and non-canonical NFκB pathways in B cells (**Figure 1**) (15, 48). It would be interesting to study whether the CD40 dose differentially activates canonical and non-canonical NFκB pathways in GC B cells and the impact of these pathways on MBC generation. The non-canonical NFκB pathway generates RelB:p52 dimer, although it could generate cRel:p50 and RelA:p50 dimers (discussed above). *In-vitro* “induced GC culture system” revealed that cRel and RelA are required for MBC generation (65). Further studies are required to identify the role of the NFκB system in the generation and reactivation of MBC subsets.

## Conclusion and future direction

With the advancement of cell type-specific conditional knockouts, we know that the NFκB system is essential for generating healthy humoral immunity, and each NFκB monomer has a unique role in the generation of GC B cells and PCs. The basic understanding of the function of the NFκB system in the regulation of GC B cell and PC generation improved our understanding of the NFκB system function in B cell pathology (B cells lymphoma, autoimmunity, and immune deficiency). However, several questions remain unanswered and need to be addressed. (1) The role of NFκB system in the generation and reactivation of MBCs and their subsets are not known. (2) Antibody-secreting cells are highly heterogenous both phenotypically and functionally. The role of NFκB system in the generation of heterogenous antibody-secreting cells are not known. (3) It is unclear how GC B cells respond to receiving multiple cell surface receptor (BCR, CD40, ICOSL, etc.) signals sequentially and combinatorially. Understanding how these signals integrate into the NFκB system and push the cell fate decision towards PC, MBC, cell division, and cell death will be interesting. Integrating mathematical modeling with experiments will be essential to understand this process.

## Author contributions

KR conceptualized the paper. KR and MC wrote the paper. AK, AM, and NR edited the paper. All authors contributed to the article and approved the submitted version.
